# Association of serum content of 25-hydroxy vitamin D with semen quality in normozoospermic and oligoasthenoteratozoospermic men

**Published:** 2018-11

**Authors:** Elham Azizi, Mohammad Naji, Maryam Shabani-Nashtaei, Aligholi Aligholi, Atefeh Najafi, Fardin Amidi

**Affiliations:** 1 *Department of Anatomy, School of Medicine, Tehran University of Medical Sciences, Tehran, Iran.*; 2 *Urology and Nephrology Research Center, Shahid Beheshti University of Medical Sciences, Tehran, Iran.*

**Keywords:** Vitamin D, Infertility, DNA fragmentation, Reactive oxygen species, Semen analysis

## Abstract

**Background:**

Vitamin D has multifaceted function in human reproductive physiology. It has been revealed that vitamin D is involved in spermatogenesis, and semen quality can be linked to vitamin D status in men.

**Objective:**

Evaluating the correlation of 25-hydroxy vitamin D (25-OHD) levels in serum with basic and advanced semen parameters and essential determinants of spermatozoa function.

**Materials and Methods:**

Participants were categorized, based on semen parameters, into normozoospermic (NS) and oligoasthenoteratozoospermic (OAT) men. Serum level of 25-OHD was measured. Apoptotic status of spermatozoa, mitochondrial membrane potential and reactive oxygen species content of semen were assessed.

**Results:**

Difference of 25-OHD concentration in serum of NS men versus OAT ones did not meet significance threshold. DNA fragmentation, reactive oxygen species content of semen and mitochondrial membrane potential state revealed significant difference between NS and OAT subjects. There were no significant differences in basic and functional semen parameters when men were stratified based on serum 25-OHD level. Taking both 25-OHD and semen categories (NS and OAT) into consideration did not indicate any significant difference in studied parameters. Total motility of spermatozoa was positively correlated with serum concentration of 25-OHD in all studied subjects. In addition, normal morphology of spermatozoa in NS men revealed a positive and significant correlation with levels of 25-OHD in serum.

**Conclusion:**

Vitamin D may affect motility and morphology of spermatozoa. Lower content of serum vitamin D may affect fertility of men and should be considered in examination of men with abnormal spermogram.

## Introduction

Vitamin D, a multi-functional signaling agent, has been revealed to mediate a broader spectrum of physiological processes than its classic effects on bone health and calcium homeostasis. Recently, multifaceted roles have been attributed to vitamin D in human reproduction (1, 2). Vitamin D utilizes two pathways to exert its effects: (i) the classical genomic pathway that acts by its binding to vitamin D receptor (VDR) and (ii) the rapid response pathway (non-genomic pathway) (3). The genomic pathway is mainly responsible for protection of cells against DNA damage, induction of cell cycle arrest, cell proliferation blockade, higher rate of apoptosis, and stimulation of differentiation (4, 5). 

On the other hand, the rapid response pathway is generally limited to protection against UV-induced DNA damage which is mainly derived by the cis-form of calcitriol (3, 6, 7). Several findings have shown that vitamin D deficiency is associated with increased risk of various disorders such as cancer, multiple sclerosis and diabetes (8). High prevalence of vitamin D deficiency has been reported in Iranian population regardless of their geographical residence in Iran (9, 10).

In men, VDR has been found to be expressed in prostate, testis, ejaculated spermatozoa and Sertoli cells (11, 12). Furthermore, expression of vitamin D-metabolizing enzymes in human testis, ejaculatory tract and mature spermatozoa has been demonstrated, which may propose significant roles of vitamin D in spermatogenesis and maturation of spermatozoa (13). It has been suggested that vitamin D-VDR binding may play role in Ca2+ dependent processes such as hyperactivated motility, capacitation and acrosome reaction (14). 

Moreover, it was shown that vitamin D could modulate cholesterol efflux, phosphorylation of tyrosine and threonine residues on specific proteins and improve sperm survival and motility (14, 15). There is a growing body of evidence regarding the association of vitamin D level with semen quality and functional significance of vitamin D (13, 14, 16-18). Jensen and colleagues studied the expression of CYP24A1, as a vitamin D-inactivating enzyme which regulates the cellular availability of vitamin D, and found a significant lower number of CYP24A1 positive spermatozoa in subfertile men versus young men with normal semen parameters, which propose CYP24A1 as a marker of semen quality (13). 

It has been illustrated that reactive oxygen species (ROS) at low levels may take part in key processes such as capacitation and acrosome reaction. However, higher levels of ROS are supposed to be associated with sperm damage and infertility in men (19). Currently, there are well-documented findings from clinical trials and animal studies confirming the indisputable role of vitamin D on reducing ROS and avoiding DNA lesions (20, 21). Sperm DNA damage is of great importance in fertility as its correlation with semen parameters and IVF results has been confirmed (22, 23). In the current study, we aimed to determine and evaluate the correlation of 25-hydroxy vitamin D (25-OHD) in serum with semen parameters, ROS content, mitochondrial membrane potential profile and sperm DNA fragmentation in normozoospermic and oligoasthenoterato-zoospermic men.

## Materials and methods

**Sampling and semen analysis**

Semen samples were collected by masturbation from men who referred to infertility clinic of Shariati Hospital, Tehran, Iran. Smoking, alcohol drinking, drug consumption, taking vitamin D containing supplements, abstinence days more than 3-5 days, history of reproductive system surgeries, urogenital infections, hormonal treatment or diseases, malignancies and systematic diseases (like renal disorders) were defined as exclusion criteria. Basic semen parameters, including sperm concentration, total motility and morphology, were evaluated after liquefaction at 37^o^C, according to World Health Organization 2010 recommendations for a cross sectional study (24). 

Participants were divided into: normozoospermic men (NS) (n=20) with normal semen parameters and no history of treatment for infertility; and oligoasthenoteratozoospermic men (OAT) (n=42) with abnormal semen parameters. All men abstained for 3-5 days before sample collection. Serum samples were collected after centrifugation and stored at -70^o^C. 25-OHD concentration in serum was determined by LIAISON–25OHD Total Assay (DiaSorin, Italy) per manufacturer’s instruction. 

Briefly, in the first incubation step, 25-OHD was dissociated from its binding protein and captured by its specific antibody, followed by addition of tracer-vitamin D linked to an isoluminol derivative and starter reagent which led to chemiluminescent flash; which was detected by a luminometer plate reader. Based on 25-OHD concentration of serum, men were categorized into: (i) vitamin D deficient (25-OHD <10 ng/ml), (ii) vitamin D insufficient (10 ng/ml ≤25-OHD ≤20 ng/ml) and (iii) vitamin D sufficient (normal level) (25-OHD >20 ng/ml) (18).

**Chemiluminescent assay of ROS in neat semen**

Freshly prepared solution (5 mM) of luminol (5-amino-2,3-dihydro-1,4-phthalazinedione, Sigma Chemical Co., St. Louis, MO, USA) in dimethylsulphoxide (DMSO, Sigma Chemical Co, USA) was mixed with neat semen and ROS level was measured using the Synergy H4 Hybrid microplate reader (BioTek, USA); semen was omitted in blank sample. Chemiluminescence signal was measured integrally for 15 min and data were analyzed by Gen5 software (BioTek, USA). Results were presented as the relative light units (RLU) per minute and per 20×10^6^ spermatozoa.

**Sperm DNA fragmentation**

DNA fragmentation was assessed by terminal deoxynucleotidyl transferase dUTP nick end labeling (TUNEL) method using Cell Death Detection Kit (Roche, Germany) according to the manufacturer’s instruction. Briefly, spermatozoa were fixed in 2% paraformaldehyde for 30 min at RT. Afterward, cells were permeabilized (0.1% Triton X-100, 0.1% sodium citrate) for 10 min on ice and incubated with TdT reaction solution containing nucleotides and TdT enzyme for 60 min at 37^o^C in the dark. TdT enzyme was omitted from negative control samples. The samples were analyzed using ﬂow cytometry (Becton Dickinson, USA) with an air-cooled argon 488 nm laser and at least 10,000 cells were detected in each group. The acquired data were analyzed by gating the dot plots and calculating TUNEL positive population using CellQuest software (Becton Dickinson, USA).

**JC-1 assay**

Mitochondrial membrane potential state was studied by JC-1 dye (Thermoscientific, USA) according manufacturer instruction. Briefly, JC-1 dye (2 µM) was added to 1 ml of spermatozoa suspension in PBS with 1×10^6^ cell/ml concentration and incubated at 37^o^C for 30 min. Afterward, the cell suspension was centrifuged and the pellet was resuspended in PBS and analyzed by ﬂow cytometry (Becton Dickinson, USA) using 488 nm laser for excitation. J-aggregates (indicator of high mitochondrial membrane potential, hMMP) were measured in FL-2 channel (585 nm) but, JC-1 monomers which indicate low mitochondrial membrane potential were measured in FL-1 channel (530 nm).

**Ethical consideration**

All sampling procedures were approved by Research Deputy and ethic committee of Tehran University of Medical Sciences (92-01-30-21270) and participants were informed and gave their written consent.

**Statistical analysis**

Data were presented as the mean±standard error. The Kolmogorov-Smirnov test was utilized to evaluate the normality of Data. T-test, Mann-Whitney U test, Kruskal-Wallis test and Spearman’s rank correlation in SPSS software (the Statistical Package for the Social Sciences, Version 18.0, SPSS Inc, Chicago, Illinois, USA) were used to compare the data and their association with 25-OHD concentration. p≤0.05 were considered as statistically significant.

## Results

**Semen analysis, DNA fragmentation, ROS content and mitochondrial membrane potential assessment**

Semen samples were evaluated according to the World Health Organization 2010 Guidelines. Samples were considered as normozoospermic when sperm count ≥15×10^6^/ml, total motility ≥40%, morphology ≥4% and Endtz test <1.0×10^6^/ml. Based on semen analysis, subjects were classified into NS (n=20) and OAT (n=42) men. No significant differences between NS and OAT subjects in age (34.1±1.2 vs. 33.02±0.7 yr, respectively) and BMI (27.3±0.4 vs. 28.06±0.3) were observed. Concentration, total motility and normal morphology of spermatozoa were significantly lower in OAT group when compared to NS subjects (Table I). 

**Figure 1:**
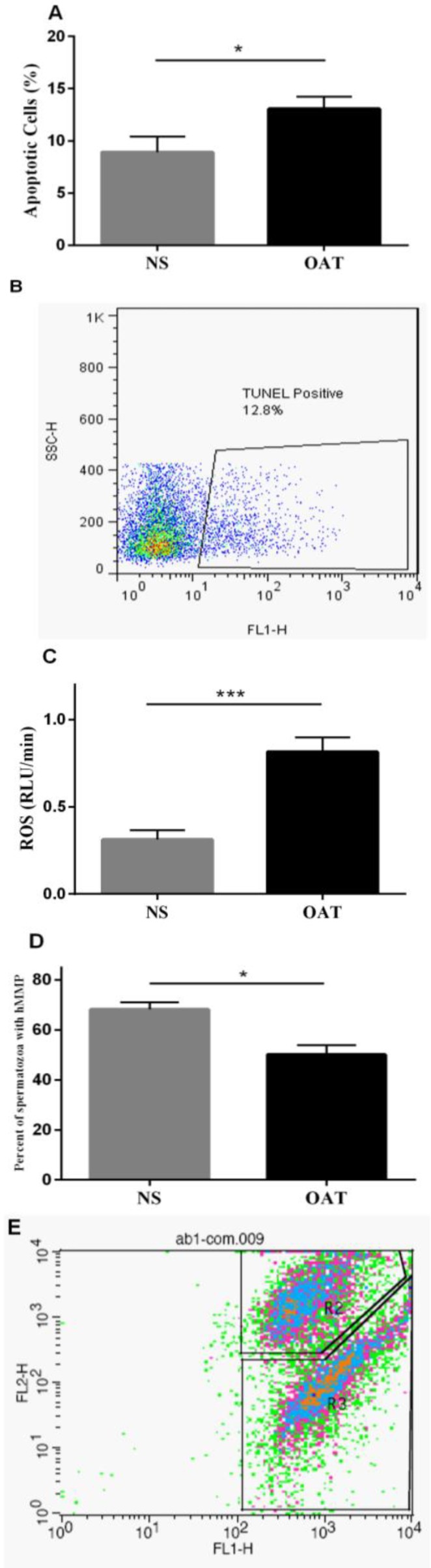
Evaluation of apoptosis, ROS content and mitochondrial membrane potential. (A) Detection of apoptotic spermatozoa by TUNEL method in NS and OAT men. (B) A representative dot plot presenting TUNEL positive gated spermatozoa. (C) Detection of total ROS in semen of NS and OAT men. Total ROS was significantly higher in OAT men versus NS men. (D) NS subjects showed greater number of spermatozoa with hMMP versus OAT subjects. (E) Representative gating of events showing spermatozoa with hMMP in R2 and low MMP ones in R3. p<0.05^*^, p<0.001^***^.

Using TUNEL assay, it was shown that TUNEL positive population of spermatozoa was significantly increased in OAT men (13.06±1.1%) compared to NS subjects (8.9±1.5%) (p=0.04) (Figure 1A). Furthermore, total ROS content was also significantly elevated in the semen of OAT men (0.81±0.08 RLU/min) compared to NS ones (0.31±0.05 RLU/min) (p=0.0004) (Figure 1B). Spermatozoa with high mitochondrial membrane potential (hMMP) comprise 68.23±2.8% of spermatozoa population in NS men while 50.13±3.7% of spermatozoa population in OAT men showed hMMP (p=0.01) (Figure 1C, D).

**Concentration of 25-OHD in serum**

The elevated level of 25-OHD in serum of NS men failed to meet significance threshold (p=0.18) (Table I). When subjects were classified according to 25-OHD level of serum, no significant differences were observed in spermatozoa concentration, total motility, normal morphology, TUNEL positive percentage, ROS content and fraction of spermatozoa with hMMP (Table II). 

However, as expected, concentration of 25-OHD in groups with insufficient and normal levels was significantly increased compared to deficient group (p<0.0001 and p<0.0001). Similarly, no significant differences were noticed when NS and OAT subgroups in each class of 25-OHD level were taken into account (Table III); except for concentration of 25-OHD which revealed significant increase in each subgroup of NS and OAT of insufficient and normal men when compared with their counterparts in deficient men. 

Total motility was positively correlated with 25-OHD concentration of serum when all subjects analyzed (p=0.27, p=0.032). In NS group, normal morphology of spermatozoa showed positive correlation with concentration of 25-OHD (p=0.53, p=0.014). However, in men with insufficient level of 25-OHD (10 ng/ml ≤25-OHD≤20 ng/ml) a negative correlation between total motility and 25-OHD was observed (p=-0.36, p=0.046).

**Table I:** Characteristics of studied men

	**Normospermic men (n= 20)**	**OAT men (n= 42)**	**p-value**
Age (yr)	34.1 ± 1.2	33.02 ± 0.7	0.41
BMI (kg/m^2^)	27.3 ± 0.4	28.06 ± 0.3	0.19
Sperm concentration (M/ml)	42.25 ± 5.7	11.18 ± 0.5	0.00
Sperm total motility (%)	51.45 ± 2.3	24.81 ± 1.6	0.00
Normal morphology (%)	11.3 ± 1	1.73 ± 0.1	0.00
25-OHD concentration (ng/ml)	15.6 ± 2.2	12.74 ± 1.3	0.18

Data were presented as mean ± standard error of mean (Mann–Whitney U test)

OAT: Oligoasthenoteratozoospermia,

BMI: Body mass index

25-OHD: 25-hydroxy vitamin D

**Table II:** Comparison of basic and functional semen parameters in different classes of 25-OHD concentration

	**25-OHD<10 ng ml** ^-1 ^ **(n= 26)**	**10 ng ml** ^-1^ **≤25-OHD<20 ng ml** ^-1 ^ **(n= 31)**	**25-OHD≥20 ng ml** ^-1 ^ **(n= 5)**
Sperm concentration (M/ml)	17.4 ± 3.9	23.55 ± 3.8	26.4 ± 8.5
Sperm total motility (%)	29.54 ± 3.5	35.84 ± 2.7	38.4 ± 5.7
Normal morphology (%)	3.34 ± 0.7	5.54 ± 1	5.2 ± 1.6
25-OHD concentration (ng/ml)	7.53 ± 0.3	15.07 ± 0.5^a^	36.8 ± 7.7^b^
TUNEL positive cells (%)	14.37 ± 1.6	9.82 ± 1.2	9.63 ± 2.1
ROS content (RLU/min)	0.79 ± 0.09	0.57 ± 0.09	0.42 ± 0.18
hMMP sperms (%)	50.76 ± 3.4	59.9 ± 4.8	70.23 ± 0.96

Data were presented as mean±standard error of mean (Kruskal-Wallis test followed by Dunn’s multiple comparison, and ANOVA followed by Tukey’s multiple comparison)

25-OHD: 25-hydroxy vitamin D

ROS: reactive oxygen species

hMMP: high mitochondrial membrane potential

a ; significant difference between insufficient and deficient groups

b ; significant difference between sufficient and deficient groups

**Table III:** Comparison of basic and functional semen parameters in NS and OAT men categorized according to concentration of 25-OHD

	**25-OHD<10 ng ml** ^-1^		**10 ng ml** ^-1^ **≤25-OHD<20 ng ml** ^-1^		**25-OHD≥20 ng ml** ^-1^	
	**NS (n= 5)**	**OAT (n= 21)**	**NS (n= 12)**	**OAT (n= 19)**	**NS (n= 3)**	**OAT (n= 2)**
Sperm concentration (M/ml)	42.8 ± 17.3	11.36 ± 0.6	43.4 ± 6.6	11 ± 0.8	36.67 ± 10.5	11 ± 3
Sperm total motility (%)	55.6 ± 8	23.33 ± 2.5	50.75 ± 2.2	26.42 ± 2.4	47.33 ± 1.4	25 ± 5
Normal morphology (%)	10 ± 1.6	1.76 ± 0.2	11.58 ± 1.4	1.73 ± 0.26	7.66 ± 1.2	1.5 ± 0.5
25-OHD concentration (ng/ml)	6.94 ± 0.8	7.68 ± 0.3	14.63 ± 0.7^a^	15.34 ± 0.7^a^	33.87 ± 8.4^a^	41.2 ± 18.9^a^
TUNEL positive cells (%)	14.09 ± 4.4	14.44 ± 1.7	7.01 ± 1.3	11.61 ± 1.6	7.82 ± 3.2	12.35 ± 1.3
ROS content (RLU/min)	0.45 ± 0.15	0.86 ± 0.11	0.24 ± 0.04	0.78 ± 0.12	0.34 ± 0.2	0.54 ± 0.43
hMMP sperms (%)	65.91 ± 5.5	48.24 ± 3.4	68.24 ± 4.07	50.53 ± 8.3	70.5 ± 1.6	69.7 ± 2.8

Data were presented as mean ± standard error of mean (Kruskal–Wallis test followed by Dunn’s multiple comparison)

25-OHD: 25-hydroxy vitamin D

NS: normozoospermic

OAT: oligoasthenoteratozoospermic

ROS: reactive oxygen species

hMMP: High mitochondrial membrane potential

a ; significant difference when compared with its counterpart in deficient group (25-OHD<10 ng ml-1)

## Discussion

In the current study, we investigated the association between serum 25-OHD concentration and semen quality in NS and OAT men. The elevated level of 25-OHD in NS men was modest. Although apoptotic status, mitochondrial membrane potential of spermatozoa and ROS content of semen were significantly different between NS and OAT men, no significant changes were observed after classification of subjects based on 25-OHD concentration. The same trend was also detected for concentration, total motility and normal morphology of spermatozoa. 25-OHD level was positively correlated with total motility and morphology of spermatozoa in all studied and NS men, respectively; but negative correlation was noticed in men with insufficient level of 25-OHD.

To the best of our knowledge, this is the first report which tried to demonstrate the association between serum levels of 25-OHD and apoptotic status of spermatozoa and ROS content of semen. ROS diminish fluidity of plasma membrane by lipid peroxidation of unsaturated fatty acids in plasma membrane of spermatozoa which consequently decline sperm function (25). It has been proposed that vitamin D can guard proteins and cell membrane from oxidative stress through preventing peroxidative insults (19). Lack of antioxidant protection, infection and production of free radicals in spermatozoa are prominent sources of oxidative stress (26). We postulated that there might be an association between serum 25-OHD and ROS of semen and DNA fragmentation of spermatozoa. Herein, we observed a tendency for reduction in apoptotic cells and ROS content with increment in 25-OHD concentration which did not reach the significance threshold. It was revealed that vitamin D could react with glutathione radicals and deactivate biological damages induced by free radicals (27). It has been shown that vitamin D in animal and in vitro studies reduced oxidative stress insults and chromosome abnormalities; it may also prevent telomere shortening and telomerase inhibition (20). The importance of sperm DNA fragmentation in fertility has been highlighted by its significant correlation with semen parameters and IVF outcome (22, 23). ROS could break DNA strands by reaction with bases. Antioxidants can prevent genetic alternations by safeguarding DNA from free radicals insult. Banakar and co-workers using hepatocellular carcinoma model in rats demonstrated that diethylnitrosamine (liver carcinogen) induced DNA damage which could be rescued with cholecalciferol treatment (4). While many of the reported findings favor protective and causative role of vitamin D in male reproductive function, there are still notable negative and irrelevant findings (28, 29). 

Frequency of vitamin D deficiency in Iranian population has been investigated in several studies and all reported a considerable high rate for vitamin D deficiency (9, 10). Rahnavard and colleagues showed that about 72% of Iranian healthy men aged from 20 to 40 yr suffered from different levels of vitamin D deficiency (9). Similarly, about 63% of subjects in our study would be categorized as vitamin D deficient if we utilize their criteria. Contrary to the report of Abbasihormozi and co-workers we observed a significantly lower proportion of men with sufficient level of serum vitamin D in our work (18).

It has been demonstrated that besides to sex hormones, vitamin D may modulate human reproduction (30). Pioneer works for uncovering the importance of vitamin D in human fertility focused on the correlation of 25-OHD level and in vitro fertilization IVF outcome which revealed significant relationship between serum content of 25-OHD and follicular fluid FF level (31, 32). Interestingly, Ozkan and co-workers showed direct correlation of vitamin D level with increased rate of clinical pregnancy and implantation (31), but another study proposed detrimental effects of elevated 25-OHD concentration on the IVF outcome (32). Regardless of opposing results, the importance of vitamin D was reinforced and more questions were raised than answered ones (31, 32). VDR and vitamin D-metabolizing enzymes have been found to be expressed in prostate, testis and germ cells (11-13). Furthermore, vitamin D complex with VDR may participate in calcium-dependent processes such as hyperactivated motility, capacitation and acrosome reaction (14). The importance of vitamin D signaling in male fertility was more underscored by VDR knockout mice in which decreased sperm counts and motility as well as testicular abnormalities caused impaired fertility (33). In this way, it was revealed that vitamin D could improve sperm survival and motility through phosphorylation of special proteins and modulation of cholesterol efflux (14, 15). The association of vitamin D concentration and sperm motility has been reported in several studies which collectively, in accordance to our findings, confirms the significant correlation of vitamin D shortage with lower sperm motility (16-18, 34-36). We also detected a positive correlation between normal morphology of spermatozoa and concentration of 25-OHD in serum of NS men. Independent studies have also demonstrated the association of 25-OHD with morphology of spermatozoa (16, 34, 35). However, total motility of spermatozoa showed negative correlation with level of 25-OHD in men with insufficient level of 25-OHD. Hammoud and colleagues have reported an inverse relationship between 25-OHD concentration in serum and motility of spermatozoa and semen quality (28). We did not detect any significant differences in 25-OHD concentration between NS and OAT subjects; in addition, concentration, total motility and morphology of spermatozoa were unchanged in different classes of 25-OHD levels. These findings are in agreement with previous reports which aimed to indicate the association of vitamin D with semen quality (16-18, 37, 38). Absence of consensus cut-off levels for serum 25-OHD may cause difficulties in interpretation of different reports. Although different from earlier studies which tried to draw a link between vitamin D concentration of serum and semen quality, we examined advanced semen parameters; the small sample size of the current study limited us from more elaborate analyses. Detection method of vitamin D has to be carefully considered in interpretation and extension of data concerning vitamin D measurements. Different methods could yield intensely unlike results from similar samples (39).

## Conclusion

Although the detailed role of vitamin D in spermatogenesis and semen quality is uncertain, more elaborative detection methods and large cohorts may be of significant help in interpretation of vitamin D function. Motility and morphology of spermatozoa may possibly be affected by vitamin D level. Owing to high occurrence of vitamin D deficiency in Iranian men, evaluation of the serum concentration of vitamin D should be considered in primary assessment of fertility in men. Vitamin D supplementation in human subjects with low semen quality should be considered and assessed in future. 

## References

[1] Anagnostis P, Karras S, Goulis DG (2013). Vitamin D in human reproduction: a narrative review. Int J Clin Pract.

[2] Blomberg Jensen M (2014). Vitamin D and male reproduction. Nat Rev Endocrinol.

[3] Haussler MR, Jurutka PW, Mizwicki M, Norman AW (2011). Vitamin D receptor (VDR)-mediated actions of 1α, 25 (OH) 2 vitamin D 3: genomic and non-genomic mechanisms. Best Pract Res Clin Endocrinol Metab.

[4] Banakar MC, Paramasivan SK, Chattopadhyay MB, Datta S, Chakraborty P, Chatterjee M (2004). 1alpha, 25-dihydroxyvitamin D~ 3 prevents DNA damage and restores antioxidant enzymes in rat hepatocarcinogenesis induced by diethylnitrosamine and promoted by phenobarbital. World J Gastroenterol.

[5] Krishnan AV, Trump DL, Johnson CS, Feldman D (2012). The role of vitamin D in cancer prevention and treatment. Rheum Dis Clin North Am.

[6] Norman AW, Mizwicki MT, Norman DP (2004). Steroid-hormone rapid actions, membrane receptors and a conformational ensemble model. Nat Rev Drug Discov.

[7] Dixon KM, Deo SS, Norman AW, Bishop JE, Halliday GM, Reeve VE (2007). In vivo relevance for photoprotection by the vitamin D rapid response pathway. J Steroid Biochem Mol Biol.

[8] Pearce SH, Cheetham TD (2010). Diagnosis and management of vitamin D deficiency. BMJ.

[9] Rahnavard Z, Eybpoosh S, Homami MR, Meybodi HA, Azemati B, Heshmat R (2010). Vitamin D deficiency in healthy male population: Results of the Iranian multi-center osteoporosis study. Iran J Public Health.

[10] Tabrizi R, Moosazadeh M, Akbari M, Dabbaghmanesh MH, Mohamadkhani M, Asemi Z (2018). High prevalence of vitamin d deficiency among iranian population: A systematic review and meta-analysis. Iran J Med Sci.

[11] Corbett ST, Hill O, Nangia AK (2006). Vitamin D receptor found in human sperm. Urology.

[12] Habib FK, Maddy SQ, Gelly KJ (1990). Characterisation of receptors for 1,25-dihydroxyvitamin D3 in the human testis. J Steroid Biochem.

[13] Blomberg Jensen M, Nielsen JE, Jørgensen A, Rajpert-De Meyts E, Kristensen DM, Jørgensen N (2010). Vitamin D receptor and vitamin D metabolizing enzymes are expressed in the human male reproductive tract. Hum Reprod.

[14] Aquila S, Guido C, Middea E, Perrotta I, Bruno R, Pellegrino M (2009). Human male gamete endocrinology: 1alpha, 25-dihydroxyvitamin D3 (1, 25 (OH) 2D3) regulates different aspects of human sperm biology and metabolism. Reprod Biol Endocrinol.

[15] Aquila S, Guido C, Perrotta I, Tripepi S, Nastro A, Andò S (2008). Human sperm anatomy: ultrastructural localization of 1α, 25‐dihydroxyvitamin D3 receptor and its possible role in the human male gamete. J Anat.

[16] Yang B, Sun H, Wan Y, Wang H, Qin W, Yang L (2012). Associations between testosterone, bone mineral density, vitamin D and semen quality in fertile and infertile Chinese men. Int J Androl.

[17] Tirabassi G, Cutini M, Muscogiuri G, Delli Muti N, Corona G, Galdiero M (2017). Association between vitamin D and sperm parameters: Clinical evidence. Endocrine.

[18] Abbasihormozi S, Kouhkan A, Alizadeh AR, Shahverdi AH, Nasr-Esfahani MH, Sadeghi Gilani MA (2017). Association of vitamin D status with semen quality and reproductive hormones in Iranian subfertile men. Andrology.

[19] Amaral S, Redmann K, Sanchez V, Mallidis C, Ramalho-Santos J, Schlatt S (2013). UVB irradiation as a tool to assess ROS-induced damage in human spermatozoa. Andrology.

[20] Nair-Shalliker V, Armstrong BK, Fenech M (2012). Does vitamin D protect against DNA damage?. Mutat Res.

[21] Chatterjee M (2001). Vitamin D and genomic stability. Mutat Res.

[22] Bounartzi T, Dafopoulos K, Anifandis G, Messini CI, Koutsonikou C, Kouris S (2016). Pregnancy prediction by free sperm DNA and sperm DNA fragmentation in semen specimens of IVF/ICSI-ET patients. Hum Fertil.

[23] Simon L, Murphy K, Shamsi MB, Liu L, Emery B, Aston KI (2014). Paternal influence of sperm DNA integrity on early embryonic development. Hum Reprod.

[24] Organization WH (2010). WHO laboratory manual for the examination and processing of human semen.

[25] Sanocka D, Kurpisz M (2004). Reactive oxygen species and sperm cells. Reprod Biol Endocrinol.

[26] Aitken RJ, De Iuliis GN (2010). On the possible origins of DNA damage in human spermatozoa. Mol Hum Reprod.

[27] Willson RL (1992). Free radical-induced biological damage and the critical roles of vitamin A, vitamin C, vitamin D and vitamin E and of copper, iron, selenium and zinc. J Nutr Sci Vitaminol.

[28] Hammoud AO, Meikle AW, Peterson CM, Stanford J, Gibson M, Carrell DT (2012). Association of 25-hydroxy-vitamin D levels with semen and hormonal parameters. Asian J Androl.

[29] Ramlau-Hansen CH, Moeller UK, Bonde JP, Olsen J, Thulstrup AM (2011). Are serum levels of vitamin D associated with semen quality? Results from a cross-sectional study in young healthy men. Fertil Steril.

[30] Lerchbaum E, Obermayer-Pietsch B (2012). Vitamin D and fertility: a systematic review. Eur J Endocrinol.

[31] Ozkan S, Jindal S, Greenseid K, Shu J, Zeitlian G, Hickmon C (2010). Replete vitamin D stores predict reproductive success following in vitro fertilization. Fertil Steril.

[32] Anifandis GM, Dafopoulos K, Messini CI, Chalvatzas N, Liakos N, Pournaras S (2010). Prognostic value of follicular fluid 25-OH vitamin D and glucose levels in the IVF outcome. Reprod Biol Endocrinol.

[33] Kinuta K, Tanaka H, Moriwake T, Aya K, Kato S, Seino Y (2000). Vitamin D Is an Important Factor in Estrogen Biosynthesis of Both Female and Male Gonads. Endocrinology.

[34] Blomberg Jensen M, Bjerrum PJ, Jessen TE, Nielsen JE, Joensen UN, Olesen IA (2011). Vitamin D is positively associated with sperm motility and increases intracellular calcium in human spermatozoa. Hum Reprod.

[35] Blomberg Jensen M, Jørgensen A, Nielsen JE, Bjerrum PJ, Skalkam M, Petersen JH (2012). Expression of the vitamin D metabolizing enzyme CYP24A1 at the annulus of human spermatozoa may serve as a novel marker of semen quality. Int J Androl.

[36] Blomberg Jensen M, Gerner Lawaetz J, Andersson AM, Petersen JH, Nordkap L, Bang AK (2016). Vitamin D deficiency and low ionized calcium are linked with semen quality and sex steroid levels in infertile men. Hum Reprod.

[37] Zhu CL, Xu QF, Li SX, Wei YC, Zhu GC, Yang C (2016). Investigation of serum vitamin D levels in Chinese infertile men. Andrologia.

[38] Tak YJ, Lee JG, Kim YJ, Park NC, Kim SS, Lee S (2015). Serum 25-hydroxyvitamin D levels and testosterone deficiency in middle-aged Korean men: a cross-sectional study. Asian J Androl.

[39] Hollis BW (2010). Assessment and interpretation of circulating 25-hydroxyvitamin D and 1, 25-dihydroxyvitamin D in the clinical environment. Endocrinol Metab Clin North Am.

